# Development of a Fault Detection Instrument for Fiber Bragg Grating Sensing System on Airplane

**DOI:** 10.3390/mi13060882

**Published:** 2022-05-31

**Authors:** Cuicui Du, Deren Kong, Chundong Xu

**Affiliations:** School of Mechanical Engineering, Nanjing University of Science & Technology, Nanjing 210094, China; 218101010101@njust.edu.cn (C.D.); dccnjust@126.com (C.X.)

**Keywords:** fault detection instrument, fiber Bragg grating sensing system, feature characteristics, synthetical anomaly index, aircraft

## Abstract

This study develops a fault detection device for the fiber Bragg grating (FBG) sensing system and a fault detection method to realize the rapid detection of the FBG sensing system on airplanes. According to the distribution of FBG sensors on airplanes, the FBG sensing system is built based on wavelength division multiplexing (WDM) and space division multiplexing (SDM) technologies. Furthermore, the hardware and software of the fault detection device and the relevant FBG demodulator are studied in detail. Additionally, in view of the similar features of the healthy FBG sensor in the same measuring point, a rapid fault diagnosis method based on a synthetical anomaly index is proposed. The features (light intensity *I*, signal length L, standard deviation of original sample σ and energy value in time-domain *P*) of FBG sensors are extracted. The aggregation center value of the above feature values is obtained through the loop iteration method. Furthermore, the separation degrees of features are calculated and then form the synthetical anomaly index so as to make an effective diagnosis of the state of the FBG sensor. Finally, the designed fault detection instrument and proposed fault detection method are used to monitor the 25 FBG sensors on the airplane, the results indicated that three faulty and two abnormal FBG sensors on the airplane are identified, showing the effectiveness of the proposed fault detection method.

## 1. Introduction

Fiber Bragg grating (FBG) sensors have been widely used in the structural health monitoring (SHM) of aircraft because of their advantages of a wide measuring range, high measuring precision and sensibility, small size, anti-electromagnetic interference, and corrosion resistance, which all make it reliable for high precision, real-time monitoring the strain, temperature and vibration of the aircraft [1,2,3].

In practical SHM aircraft engineering, a large number of FBG sensors are typically installed on the key components of aircraft (e.g., wings, tails, frame, etc.) to obtain complete and comprehensive physical information (e.g., strain, temperature, etc.) for effective structural component monitoring [4,5,6]. Furthermore, it is well known that FBG multiplexing technology is commonly used to connect a number of FBG sensors in series, which could share the same light source and demodulation system [7,8]. It also reduces the size of the network circuit and improves space usage [9]. Presently, the common multiplexing technologies such as the wavelength division multiplexing (WDM), time-division multiplexing (TDM), space division multiplexing (SDM), and frequency division multiplexing (FDM) [10,11,12]. In the WDM technology, FBG sensors with different central wavelengths are connected in series. It reduces work quantity but decreases the broadband light source power, which fails to meet the multipoint and distributed sensing measurements [13]. All FBG sensors are connected to an optical switch used to control and select the transmission channel in the SDM technology. Nevertheless, the optical switch transformed among extensive FBG sensors will affect the sampling frequency [14,15]. The separate WDM, SDM, or TDM cannot meet the demand for the SHM on aircraft.

The accuracy of FBG sensor data is of great significance to the assessments of the measuring structure of the key airplane [16]. However, the FBG sensor’s faults or failures become more frequent than the structural damage during the sensor lifetime owing to their own natural aging, line failure, installation failure, harsh operational environment (e.g., temperature, humidity and moisture), and so on. Furthermore, the sensor detection using the one-by-one method will take a long time and increase the maintenance costs, especially in the large sensor network [17,18]. The early identification of faults or failures in FBG sensors is the most important aspect of fault detection to ensure data accuracy and reliability [19,20]. Therefore, it is essential to find a rapid and effective sensor fault or failure detection method to ensure high-precision results in aircraft SHM systems. In some literature [21,22,23], the available measurements and output of the observer are compared and then the differences between them are exploited to further identify the malfunctions. The effectiveness of this method is dependent on the accuracy of the observer. CAZZULANI G. et al. [24] proposed a sensor fault identification algorithm based on the analysis of the residuals of the measurement estimation, which could identify different typologies of sensor fault or malfunctioning. In [25], a technique to assess the reliability of FBG sensors was proposed. However, it requires an expensive instrument and it is also hard to apply online. Rama Mohan Rao A. et al. [26] presented a sensor fault detection and isolation technique based on the null subspace method. An experimental test was carried out using a scaled-down model of a bridge, which clearly indicated the effectiveness of the proposed algorithm. Huang H. B. et al. [27] presented a sensor-fault detection and isolation method based on a statistical hypothesis test and missing variable approach and its performance was validated and demonstrated on the bridge health monitoring. However, the proposed method is effective in detecting and isolating both bias and drift sensor faults and is not suitable for fault detection on FBG sensors. In [28], a deep learning-based method was proposed for damage identification of the operational functionality of the smart interface device. While the method has shown promising prospects for electro-mechanical impedance (EMI)-based damage detection, it has limitations on other fields. Hence, developing and designing a fault detection device for the FBG sensing system, as well as a fault detection method to detect and maintain FBG sensors and optical fiber demodulator on an airplane is critical. Therefore, in this study, we develop an FBG sensing system based on the WDM and SDM technology, design a fault detection device with the matched FBG demodulator equipment based on the FBG sensing system, propose a wavelength demodulation method based on the volume phase grating (VPG) and linear array photodetector and a Gaussian curve fitting method for finding the peak value of the reflection spectrum. We also propose a fault detection method based on a synthetical anomaly index. The parameters (light intensity *I*, signal length L, standard deviation of original sample σ and energy value in time-domain *P*) of FBG sensors are selected as the signal features. The separation degrees of these features are obtained and merged into the synthetical anomaly index for rapidly detecting the state of FBG sensors. Finally, the proposed method is validated and demonstrated for the 25 FBG sensors on an airplane, which proves the effectiveness and good guidance in making quick and effective decisions for the state of FBG sensors.

## 2. Fiber Bragg Grating Sensing System on the Airplane

**Figure 1:**
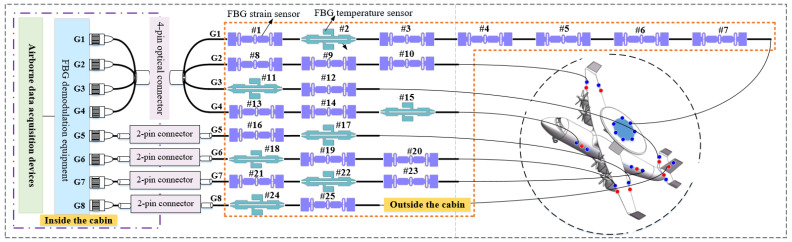
Diagram of the FBG sensing system on the plane.

FBG strain sensors with advantages of small size, wide measuring range, high measuring precision and sensibility and anti-electromagnetic interference have made them increasingly adopted as an important strain measurement for the aircraft. In Figure 1, 25 FBG sensors (18 FBG strain sensors and 7 FBG temperatures) are installed in different locations of the aircraft. The FBG sensors 1–7, 8–10, 11–12, 13–15, 16–17, 18–20, 21–23, and 24–25 are installed on the Dome, right wing, right tail, left fuselage, left wing, and vertical tail of an aircraft, respectively. The FBG strain sensors are used to measure the strain variation of the relevant measuring points, which could provide an important basis for identifying the status of the aircraft.

As shown in Figure 1, the FBG sensing system on the airplane is designed using WDM and SDM technologies. All FBG sensors on an airplane are divided into eight channels, as shown in Figure 1. A certain number of FBG sensors with different central wavelengths are connected in series in one channel through WDM technology. Each FBG sensor is assigned a bandwidth to ensure that the wavelength shift is within the bandwidth. Using SDM technology, the optical switch array in FBG demodulation equipment switches through different channels. Note that the FBG temperature sensors are used to make the temperature compensation for the FBG strain sensors. The FBG sensors, which are installed outside the cabin, are connected to the optical fiber demodulation equipment in the cabin through the cabin optical connector. Furthermore, the airborne data collector is used to collect and save the demodulation information from the FBG demodulation equipment. The combination of WDM and SDM technologies could realize the distributed measurement of multipoint measurement and ensure the FBG sensor system’s lightweight and less optical cable.

## 3. System Design and Implementation

### 3.1. Hardware Design of Fault Detection Instrument

**Figure 2:**
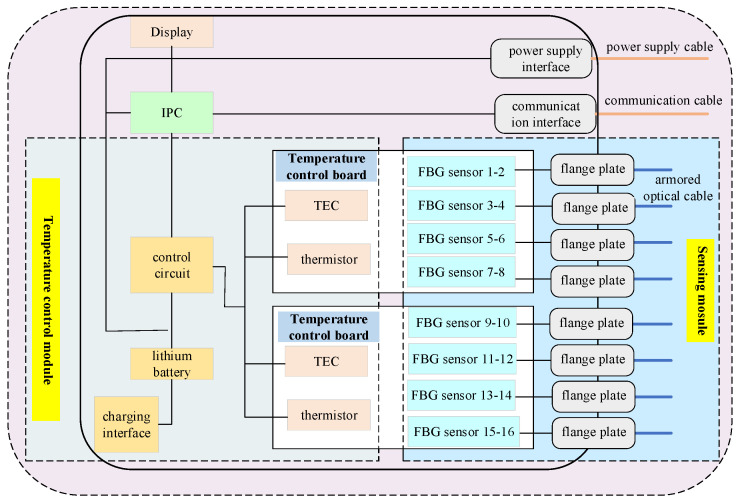
Hardware architecture of the fault detection instrument for FBG sensing system.

The fault detection device of the FBG sensing system mainly includes a lithium battery, industrial personal computer (IPC), temperature control module, FBG sensing module, power supply interface, communication interface, display, etc., as shown in Figure 2. A fault detection device is usually used with the FBG demodulator.

The fault detection device of the FBG sensing system is powered by a lithium battery, which also provides power to the IPC, control circuit, and FBG demodulator through the power supply interface. The FBG demodulator’s output data are transmitted to the IPC via a communication interface, and the upper computer software processes the data. The output optical cable of the FBG sensors is connected to the flange plate located at the device’s front panel, and 16 FBG temperature sensors are pasted on the sensing module. The armored optical cables are equipped with FC/APC optical connectors at both ends. One end of the optical cable is connected to the outside of the flange plate. The other is actualized to connect the external FBG demodulator to realize the conduction of the optical path.

A temperature control module comprises a temperature control board (cold board and hot board), thermoelectric cooler (TEC), thermistor, and control circuit. TEC, thermistor, and the FBG temperature sensors are all pasted on the surface of the cold board. A thermistor is used to perceive the temperature change of the cold board, convert the change into an electrical signal, and then transmit the signal to the control circuit. The control circuit algorithm is used to control the TEC or thermistor to achieve a relatively constant temperature of the cold board and further make the FBG temperature sensors enhance a stable output. Note that the strain induced by the external force or other factors will not affect the performance of the FBG temperature owing to its mounting ear structure. When the temperature control module’s temperature accuracy reaches 0.1 °C, the installed FBG temperature sensor can achieve a temperature sensitivity of 0.028 nm/°C, and its wavelength shift can be controlled within 3 pm.

**Figure 3:**
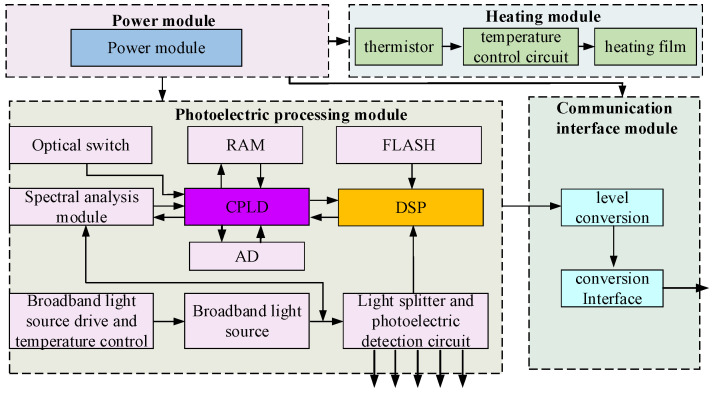
Hardware implementation of the matched FBG demodulation equipment.

The function of the FBG demodulation equipment is used to make the sensing information detected by the FBG sensors demodulated in the form of the wavelength encoding. The external FBG demodulator equipment is typically used with a designed fault detection device when the FBG sensors on an airplane need to be detected. Hence, the matched FBG demodulation equipment is also designed, as shown in Figure 3. The FBG demodulation equipment is mainly composed of a power module, photoelectric processing module, communication interface module and heating module. The heating module provides a heat preservation function for the spectral analysis module and an optical switch circulator.

As for the photoelectric processing module, the broadband light source that is driven by a constant current circuit could provide the broadband light with a wavelength range from 1510 nm to 1590 nm. The photoelectric detection circuit is mainly used to monitor the light intensity value of the laser and give feedback to the control circuit so that the light intensity value can be adjusted. The output light enters the optical switch through the circulator and the reflected light from the FBG sensor enters the spectral analysis module which is used to convert the light signal into a pixel voltage signal. The voltage signal is converted to digital quantity through an AD converter. A complex programmable logic device (CPLD) realizes the control function and transmits the spectral data to digital signal processing (DSP) through the communication interface. The central wavelength of the FBG sensor was fitted and calculated in DSP. In the spectral analysis module, spectrum detection and demodulation technology based on the volume phase grating (VPG) and linear array photodetector is adopted. The VPG spectrum module with the advantages of small size, without the piezoelectric materials and moving parts, is applicable in the aircraft with large vibration situation.

### 3.2. Software Design of Fault Detection Device

**Figure 4:**
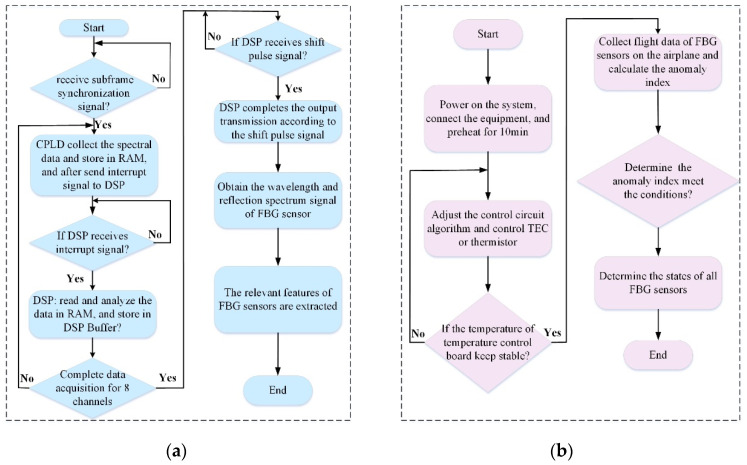
Program flowchart of the fault detection instrument. (**a**) FBG sensors; (**b**) FBG demodulation equipment.

The program flowchart of the fault detection instrument for the FBG sensing system is shown in Figure 4. Figure 4a shows the detection process of the FBG sensors. As the system has been initialized, the CPLD is in waiting for the subframe synchronization signal sent by the data acquisition unit. It receives the signal and starts the spectral signal acquisition, as well as provides the relevant driving clock in accordance with the spectral analysis module’s timing requirements. The collected data include pixel voltage signal and internal temperature signal of the spectral analysis module, and the two types of signals are switched over through an optical switch. The spectral analysis module includes 512-pixel units and all need to be collected and stored in RAM.

The CPLD then sends the interrupt signal to DSP. If DSP receives the interrupt signal and begins solving the collected data and further stores the results in the DSP buffer. The DSP then sends the command to the CPLD to begin to make data acquisition from the next channel until it completes the data acquisition of eight channels Furthermore, the DSP could sort out the data of eight channels according to the requirement of the data format and then wait for the shift pulse signal from the data acquisition unit. Once the DSP receives the shift pulse signal, then it completes the data transmission through the communication interface. Till now, the wavelength data and reflection spectrum signals of FBG sensors have been obtained. Finally, the performance of FBG sensors in the normal state could be observed and compared to the central wavelength or light intensity.

Figure 4b shows the FBG demodulation equipment detection process after the system is powered on and connected, the control circuit algorithm is used to control the TEC or thermistor until the temperature of the temperature control board achieves a steady state. Flight data is collected and the anomaly index of FBG sensors on the airplane according to the extracted features is calculated. The performance of the FBG demodulation instrument is reflected by the connected FBG sensors. Hence, the state of the FBG demodulation instrument is validated by the 16 FBG sensors in the fault detection instrument or the states of FBG sensors on the aircraft.

## 4. Wavelength Demodulation and Fault Detection Methods

### 4.1. Wavelength Demodulation Method

**Figure 5:**
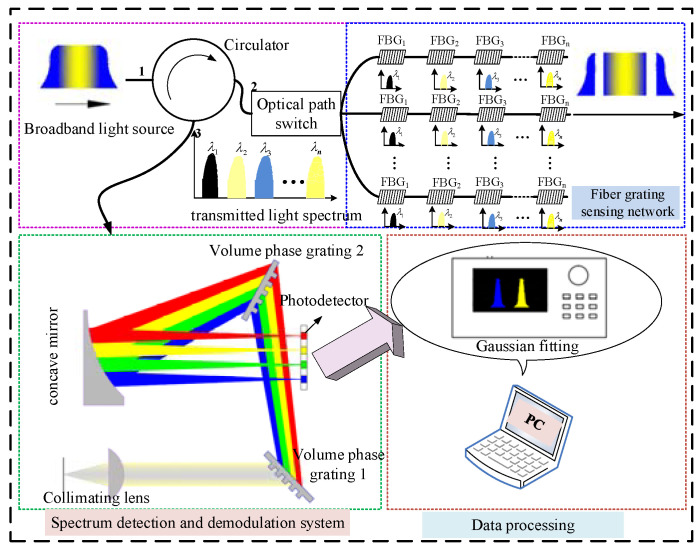
Wavelength demodulation principle and peak searching method.

As shown in Figure 5, when light from the broadband light source (BBS) is incident on the fiber grating sensor network through the circulator, the light that meets the Bragg conditions is reflected and subsequently enters the spectrum detection and demodulation system through the circulator. The optical path switch is used to convert the different channels’ optical signal transmission. The wavelength demodulation method is based on a VPG and a linear array photodetector. The reflected light source becomes the parallel light after passing through the collimating lens, which is irradiated on the VPG1. Due to the light splitting effect of the VPG, the light with different wavelengths is separated into the light with different refraction angles. Light beams with different refraction angles are converged on the concave mirror and then transmitted to different positions of the linear array photodetector. The variation in ambient temperatures of the FBG sensor will change the period and the effective refractive index of FBG, resulting in a wavelength shift of the grating signal. Furthermore, the image position on the linear array photodetector will also change accordingly.

The pixel voltage signals of the linear array photodetector are collected and processed to obtain the central wavelength shift of the fiber grating sensor. The relationship between the number of pixels and wavelength is expressed as [29]:

(1) $$\lambda =A+B_{1}pix+B_{2}pix^{2}+B_{3}pix^{3}+B_{4}pix^{4}+B_{5}pix^{5}$$

where, *pix* is the number of pixels and λ is the wavelength of the FBG sensor. *A*, *B*_1_, *B*_2_. *B*_3_, *B*_4_ and *B*_5_ are the polynomial fitting coefficients.

Equation (1) is obtained through the least squares method which is used to complete the fifth-order polynomial fitting between λ and pix. Then, the polynomial fitting coefficients are determined: *A* = 1.595306 × 10^3^, *B*_1_ = −1.355909 × 10^−1^, *B*_2_ = −6.160845 × 10^−5^, *B*_3_ = −3.346493 × 10^−11^, *B*_4_ = −1.224188 × 10^−11^, and *B*_5_ = 1.133598 × 10^−14^.

The measurement accuracy of the FBG sensor is largely dependent on the finding peak degree of the central wavelength. It is generally known that the shape of the Gaussian curve is extremely similar to the reflection spectrum of FBG [30]. To improve the FBG sensor detection accuracy, the obtained reflection spectrum signal is made denoizing and the Gaussian curve fitting method is used for linear regression to further obtain the central wavelength of the FBG sensor. The Gaussian fitting formula is given by:

(2) $$I(\lambda )=I_{0}\exp[=4\ln2(\frac{\lambda -\lambda_{s}}{\Delta \lambda_{s}})]$$

where, $I_{0}$ is the peak value of reflection spectrum intensity, $\lambda_{s}$ is the wavelength value when the reflection spectrum intensity is $I_{0}$. $\Delta \lambda_{s}$ is the half-width of reflection spectrum intensity.

### 4.2. Fault Detection Method

(a) Fault detection principle

The main load affected by FBG sensors is stress during the flight of the aircraft. The variation of loads on the same monitoring structure point is roughly similar. Furthermore, it was found that the parameters of output signals (e.g., the light intensity *I*, wavelength amplitude et al.) of the healthy FBG sensors have a similarity. Moreover, the multiple statistical features of a healthy FBG sensor are in an aggregation state. When the FBG sensor fails, the features will cause change and tend to be in a separated state. Therefore, the separation degree of the related features of the relevant measuring points could be obtained to determine the state of the FBG sensors. The common fault types and relevant signal characteristics are listed in Table 1.

(b) Feature extraction of FBG sensor

Generally, the signal statistical characteristics of the FBG sensor mainly include the light intensity, signal length, standard deviation of the original sample, energy value in the time-domain, amplitude value and variance, etc. According to the actual function of the signal, the first four parameters of the above characteristics are selected as the features for fault detection on FBG sensors. The definition of the four feature parameters is shown in Table 2.

The linear array photodetector’s measurement range is 1.6 V–3.8 V; thus, if the minimum light intensity *I_min_* is close to or equal to 1.6 V, the FBG sensor is in an abnormal or fault state. When the data acquisition device collects the data of FBG sensors on an airplane, the wavelength data that exceeds a certain threshold will be discarded. Thus, the inconsistent length L could indicate an abnormal or faulty state of the FBG sensor. σ could reflect the dispersion degree of the data set, and it is also the basis for judging the signal stability of measuring points. *P* could reflect the fluctuation intensity of the output signal, and it has obvious indications on the FBG sensor with excessive gain or no obvious change in output amplitude. The feature characteristics of *m* FBG sensors are sequentially extracted, and the feature vector is obtained as follows:

(3) $$f=\left[\begin{array}{cccc} I(1) & L(1) & \sigma (1) & P\left(1\right)\\ I(2) & L(2) & \sigma (2) & P\left(2\right)\\ \vdots & \vdots & \vdots & \vdots \\ I(m) & L(m) & \sigma (m) & P\left(m\right) \end{array}\right]$$

### 4.3. Fault Detection Process

**Figure 6:**
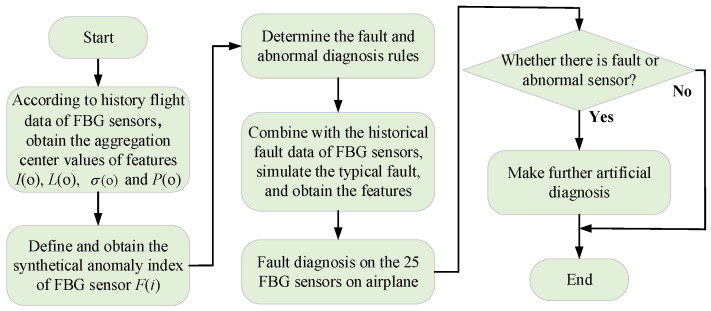
Fault detection process of FBG sensors on the airplane.

The FBG sensor fault detection process is shown in Figure 6. First, to obtain the separation degree of features, the aggregation center values of four features (*I*, *L*, σ, *P*) must be acquired. To avoid the effect of the too large separation values on the aggregation center values, the loop iteration method is used to search the feature point with the largest number of aggregation points nearby. The aforementioned feature point is regarded as the aggregation center value of the relevant feature.

The data aggregation coefficient is defined by:

(4) $$C(X)=\frac{a}{l}\sum_{b=1}^{l}\left|X(b)-\bar{X}\right|,\;(X=I,\;L,\;\sigma ,\;P)$$

where, *l* is the length of the relevant feature X(b) and $\bar{X}$ represent the four feature data and their own mean value, respectively, and *a* is a multiple of the data aggregation coefficient.

Furthermore, according to Equation (5), the Euclidean distance between two different feature points is calculated through the loop iteration method to find the aggregation center values of four features.

(5) d(i,j)=|X(i)−X(j)|

where, *i* and *j* are the serial numbers of different measuring points.

Determine the d(i,j) and C(X) whether it meets the condition d(i,j)<C(X); if yes and it shows that the *j*th value is aggregated near the *i*th value. Calculate the Euclidean distance between *i*th point and all other points until finding the point with the largest number of aggregation points near the aggregation center values of four features (*I*, *L*, σ, *P*) are called *I*(o), *L*(o), σ(o), and *P*(o), respectively.

Second, the Z-score data standardization method is used to achieve standardization for the offset distance between feature values and their aggregation center value when calculating the separation degree of features. The FBG sensor’s synthetical anomaly index E(i) is defined as follows:

(6) $$E(i)=\sum_{X}\frac{\left|X(i)-X(o)\right|}{\sigma_{X}},\;(X=I,\;L,\;\sigma ,\;P)$$

(7) $$\sigma_{X}=\sqrt{\left\{\sum_{i=1}^{l}{\left[X(i)-\bar{X}\right]}^{2}\right\}/l},\;(X=I,\;L,\;\sigma ,\;P)$$

where, $\sigma_{X}$ is the standard deviation of the relevant feature vector.

Finally, the fault and abnormal diagnostic rules are described as:

(8) $$R[E(i)]=\left\{ \begin{array}{ll} E(i)\leq t_{1}\cdot \bar{E}, & \mathrm{healthy}\;\mathrm{sensor}\\ t_{1}\cdot \bar{E}<E(i)\leq t_{2}\cdot \bar{E}, & \mathrm{abnormal}\;\mathrm{sensor}\\ E(i)>t_{2}\cdot \bar{E}, & \mathrm{faulty}\;\mathrm{sensor} \end{array}\right.$$

where, $\bar{E}$ is the mean value of the anomaly index of all measured FBG sensors. $t_{1}$ and $t_{2}$ are the anomaly coefficient and faulty coefficient, respectively.

## 5. Experimental Verification and Discussion

To evaluate the performance of the designed fault detection device and the proposed fault detection based on the FBG grating sensing system, a typical fault simulation experiment of FBG sensors and the detecting experiments of FBG sensors on an airplane perform, respectively.

### 5.1. Typical Fault Simulation Test

**Figure 7:**
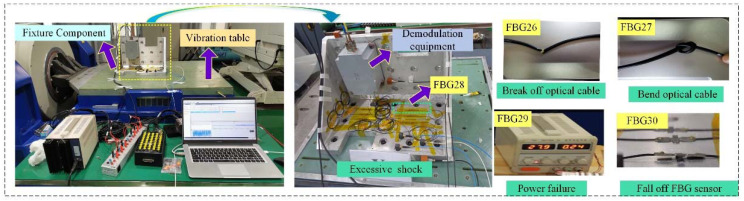
Simulation setup of typical faults on FBG sensors.

The flight data from 25 FBG sensors (the number codes are 1–25) with the healthy state on an airplane are selected as the healthy sample (Sample length of each sensor: 289,081). Additionally, the typical faults of FBG sensors are simulated, respectively. The simulation setup of typical faults on FBG sensors is shown in Figure 7.

**Figure 8:**
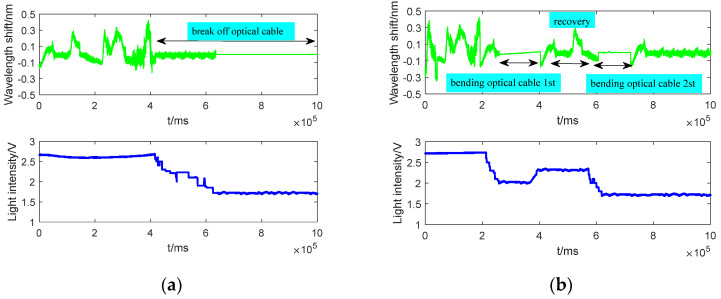

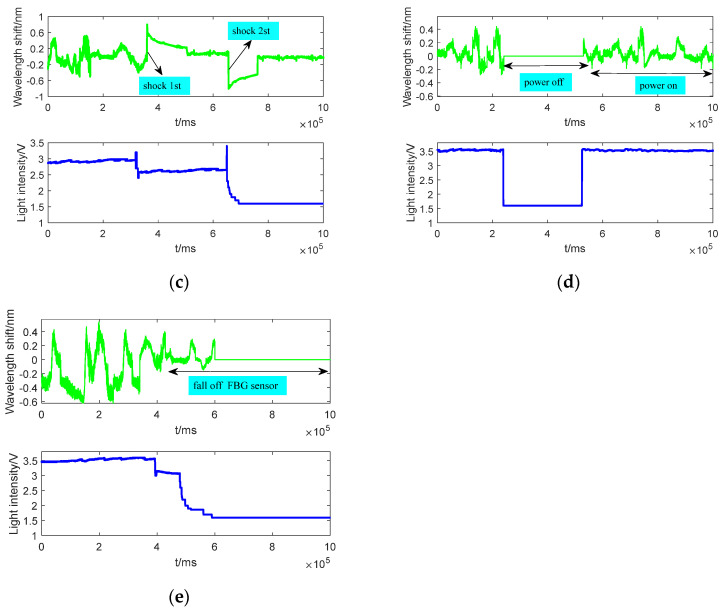
Wavelength and light intensity variation curves of five typical faults. (**a**) Break off the optical cable. (**b**) Bending the optical cable. (**c**) Shock test. (**d**) Sudden power off and power on. (**e**) Fall off FBG sensor.

According to the installation technology and the working environment of the FBG sensors on the airplane, five FBG sensors (relevant number codes are 26–30) are pasted on the fixture component with the same material as the measuring point structure of the airplane. As shown in Figure 7, the fixture component is installed on the vibration table, which provides the random vibration for the five FBG sensors. Figure 8 shows five typical faults of FBG sensors (26: break off optical cable, 27: excessive bend optical cable, 28: excessive shock, 29: sudden power failure, and 30: fall off FBG sensor) are simulated, and their partial wavelength and light intensity variation curves.

**Figure 9:**
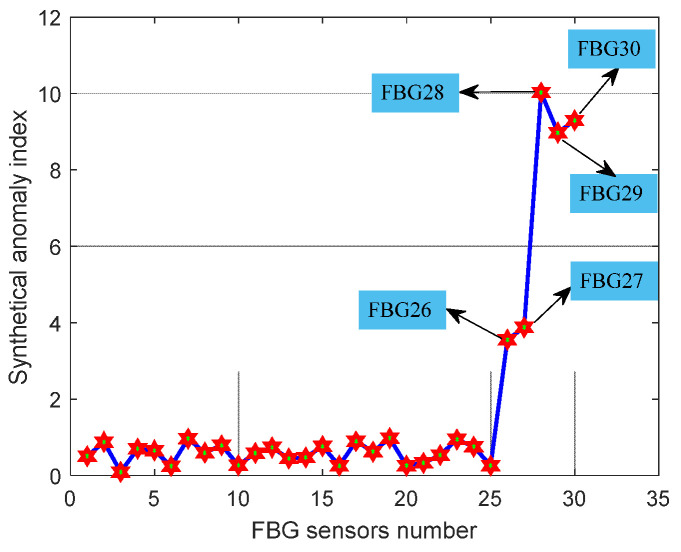
Fault detection results of 30 FBG sensors.

As shown in Figure 8a–e that the wavelength shifts and light intensities of the FBG sensors cause obvious changes when the faults occur. The data of 25 healthy FBG sensors on an airplane and the above five faulty sensors are regarded as test data. The length of each simulated fault data was equal to the data of each healthy FBG sensor. Faulty sensors using the proposed fault detection method. The features and synthetical anomaly index of faulty FBG sensors are listed in Table 3, and the fault detection results of 30 FBG sensors are clearly shown in Figure 9.

As shown in Figure 9 that the synthetical anomaly index of faulty 26, 27, 28, 29, and 30 FBG sensors is significantly higher than that of the other normal 25 FBG sensors. Furthermore, according to Equation (8) and Figure 9, we can know that too many abnormal or faulty points could cause excessive discrete distribution of features, leading to poor detection results. Then, the anomaly and faulty coefficients $t_{1}$ and $t_{2}$ are bound to make further adjustments according to the discrete degree of features.

### 5.2. Fault Detection on 25 FBG Sensors on Airplane

**Figure 10:**
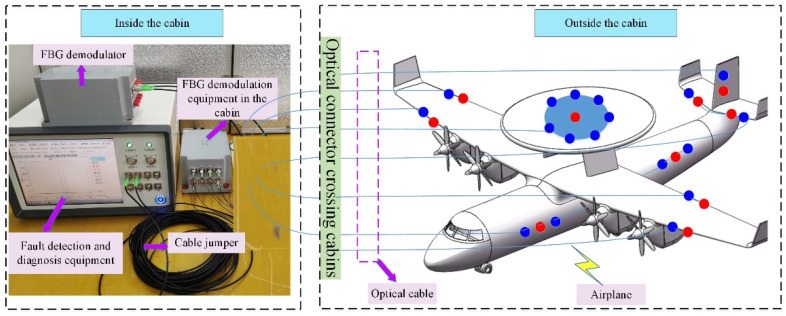
Experiment setup diagram of fault detection instrument for the FBG sensing system on an airplane.

**Figure 11:**
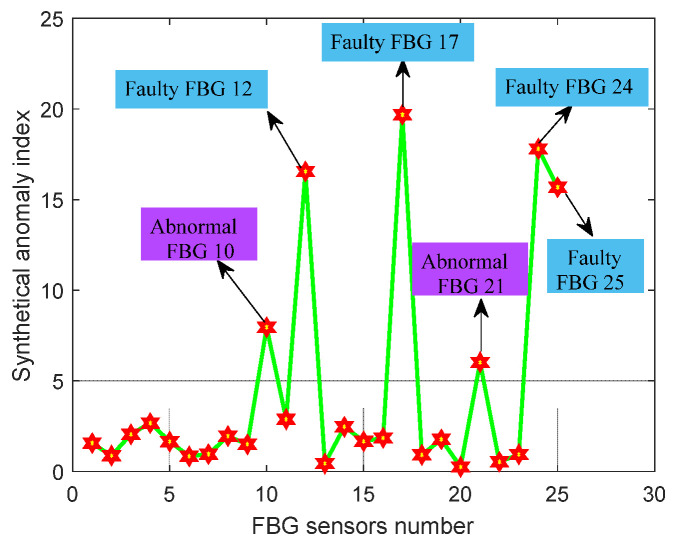
Fault detection results of 25 FBG sensors on airplane.

The test sample is flight data from 25 FBG sensors on an airplane (from November 2021 to December 2021). As shown in Figure 10, the designed fault detection instrument and the matching FBG demodulator are placed in the cabin. Through an optical connector, 25 FBG sensors installed on the key structures of an airplane are connected to the fault detection device in the cabin. The measuring data of 25 FBG sensors were extracted from the historical database, and the relevant features of all sensors were also calculated. The fault detection results of 25 FBG sensors are shown in Figure 11, and the features and synthetical anomaly index of faulty and abnormal FBG sensors are shown in Table 4.

**Figure 12:**
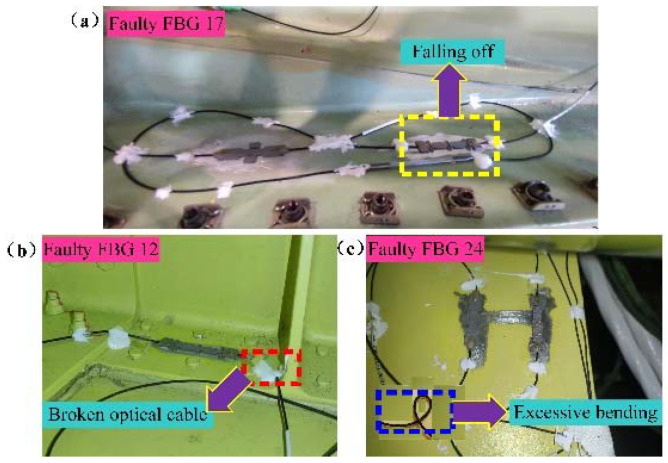
Diagram of faulty FBG sensors on airplane. (**a**) Falling off; (**b**) Broken optical cable; (**c**) Excessive bending.

As shown in Figure 11, the proposed fault detection method detects four faulty and two abnormal FBG sensors. After making further manual verification on the above faulty and abnormal sensors, it was found that the optical cable near the FBG 12 sensor had broken, FBG 17 sensor had fallen off the measured point, and the optical cable of the FBG 24 sensor was excessively bent, as shown in Figure 12a–c, respectively. The FBG 24 and 25 sensors were connected in one optical cable, and the bent optical cable also caused the abnormal measuring signal. Furthermore, the two abnormal FBG sensors (FBG 10 and 21) were affected by the high-frequency signals, which caused many abrupt changes in signals and then further generated abnormal feature values. The above experimental results show that the designed fault detection device and proposed fault detection method have better guidance in making quick and effective decisions for the states of FBG sensors on an airplane. According to the detection results, the maintenance worker would make a further diagnosis and then troubleshoot the faulty or abnormal sensors immediately and effectively. Furthermore, it is also of great significance to improve measuring data accuracy.

## 6. Conclusions

The major contribution of this research is to develop and design a fault detection device and propose a fault diagnosis method based on a synthetical anomaly index. In this study, we built an FBG sensing system based on WDM and SDM technologies based on the FBG sensor network distribution on the aircraft. Furthermore, the hardware and software of the fault detection device and the matched FBG demodulator are illustrated in detail. The simulation test and verification experiments all proved the effectiveness and good guidance in making quick and effective decisions for the states of FBG sensors. The designed fault detection device has the abilities of data acquisition, data storage, data display, and data printed in both text format and image format. It is also easy to operate and could efficiently and quickly detect the state of the FBG sensor and FBG demodulation equipment on an airplane, which could shorten the detection time, reduce the maintenance cost, and provide an important basis for the SHM of the critical structures of the aircraft. Furthermore, the proposed fault detection method could provide reliable detection results for FBG sensors without prior knowledge. However, the major limitation of the fault detection method is that the users determine the concrete fault of the relevant FBG sensor, and it needs further research.

## Figures and Tables

**Table 1:** Features values and synthetical abnormal index of five faulty FBG sensors.

Fault Type	Signal Characteristics
Damage or fall off	No change in wavelength and intensity or little change
Excessive shock	Abrupt change of signal amplitude or missing
Break off optical cable	No signal output
Excessive bend optical cable	Weaken the signal or small amplitude variation
Sudden power failure and recover	Data missing and data length reduction

**Table 2:** Definitions of four feature parameters.

Feature Parameters	Definition
Minimum light intensity *I*	$I_{\min}=\min[I(n)]$
Signal length *L*	$L=length(\lambda_{n})$
Standard deviation of original sample *σ*	$\sigma =\sqrt{\left\{\sum_{n=1}^{N}{\left[\lambda (n)-\bar{\lambda }\right]}^{2}\right\}/N}$
Energy value in time-domain *P*	$P=\sum_{n=1}^{N}{\left[\lambda (n)-\bar{\lambda }\right]}^{2}$
$\mathrm{Here},\;\left\{\lambda_{n}\right\}(n=1\sim N)$ is the original wavelength data of FBG sensor (*N* is data points)	

**Table 3:** Features values and synthetical abnormal index of five faulty FBG sensors.

FBG Sensors Number	Light Intensity *I*/V	Signal Length *L*	Standard Deviation *σ*/nm	Energy Value *P*/nm^2^	Synthetical Anomaly Index
FBG26	1.6	267,341	0.0718	5361.538	3.556338
FBG27	1.6	289,081	0.0808	6781.024	3.881325
FBG28	1.6	289,081	0.2482	34,049.181	10.02711
FBG29	1.6	192,679	0.0874	2741.718	8.97647
FBG30	1.6	227,828	0.1883	36,879.550	9.296857

**Table 4:** Features values and synthetical abnormal index of faulty and abnormal FBG sensors.

FBG Sensors Number	Light Intensity *I*/V	Signal Length *L*	Standard Deviation *σ*/nm	Energy Value *P*/nm^2^	Synthetical Anomaly Index
FBG 10	2.56	403,581	0.2708	6985.123	7.9565
FBG 12	1.60	257,562	0.1280	4909.545	16.5480
FBG 17	1.60	403,581	0.2035	4293.836	19.6785
FBG 21	2.79	403,581	0.0946	7290.521	6.0214
FBG 24	1.65	327,890	0.1023	5031.056	17.7976
FBG 25	1.71	403,581	0.1245	4986.301	15.6780

## Data Availability

The data presented in this study are available in Table 3 and Table 4 in this article.
